# Depriving Mice of Sleep also Deprives of Food

**DOI:** 10.3390/clockssleep4010006

**Published:** 2022-02-11

**Authors:** Nina Đukanović, Francesco La Spada, Yann Emmenegger, Guy Niederhäuser, Frédéric Preitner, Paul Franken

**Affiliations:** 1 Center for Integrative Genomics, University of Lausanne, 1015 Lausanne, Switzerland; nina.dukanovic@unil.ch (N.Đ.); francesco.laspada@unil.ch (F.L.S.); yann.emmenegger@unil.ch (Y.E.); 2 Mouse Metabolic Evaluation Facility, Center for Integrative Genomics, University of Lausanne, 1015 Lausanne, Switzerland; guy.niederhauser@unil.ch (G.N.); frederic.preitner@unil.ch (F.P.)

**Keywords:** circadian rhythms, clock genes, sleep deprivation, food intake, energy expenditure, body composition, lean body mass, fat

## Abstract

Both sleep-wake behavior and circadian rhythms are tightly coupled to energy metabolism and food intake. Altered feeding times in mice are known to entrain clock gene rhythms in the brain and liver, and sleep-deprived humans tend to eat more and gain weight. Previous observations in mice showing that sleep deprivation (SD) changes clock gene expression might thus relate to altered food intake, and not to the loss of sleep per se. Whether SD affects food intake in the mouse and how this might affect clock gene expression is, however, unknown. We therefore quantified (i) the cortical expression of the clock genes *Per1*, *Per2*, *Dbp*, and *Cry1* in mice that had access to food or not during a 6 h SD, and (ii) food intake during baseline, SD, and recovery sleep. We found that food deprivation did not modify the SD-incurred clock gene changes in the cortex. Moreover, we discovered that although food intake during SD did not differ from the baseline, mice lost weight and increased food intake during subsequent recovery. We conclude that SD is associated with food deprivation and that the resulting energy deficit might contribute to the effects of SD that are commonly interpreted as a response to sleep loss.

## 1. Introduction

One presumed important adaptive advantage of an endogenous, circadian time-keeping system is that it enables the organism to anticipate the daily changes in the environment such as the availability of food. The circadian system thus ensures that the animal is awake and active before food becomes available and, along with other constraints, defines the animal’s temporal ecological niche. In conflict experiments, in which access to food is restricted to a time of day when the animal is normally asleep and does not engage in feeding behavior, such as during the light period in the case of a nocturnal rodent, the animal’s behavior and physiology must adjust to anticipate the new feeding regimen. Studies in mice and rats show that these feeding-related adjustments are not immediate, and it can take several days until variables such as wakefulness and corticosterone production become entrained and peak prior to the new time of food availability [1,2,3,4]. This entrainment is also evident at the molecular level. In the cortex and liver, the phase of the oscillation in the expression of circadian clock genes adjusts to that of the new feeding time, thereby dissociating from clock gene rhythms in the central circadian pacemaker in the suprachiasmatic nuclei (SCN), which remain entrained to the light–dark cycle [3,5,6].

The expression of clock genes is also modified when sleep is altered suggesting that these genes also play a role in the homeostatic aspect of sleep regulation [7]. For instance, keeping animals awake at a time of day when they normally sleep affects clock gene expression in tissues peripheral to the SCN, including the cerebral cortex, liver, and kidney [7,8,9,10,11]. Because clock gene expression responds to alterations in both feeding and sleep-wake behavior, it has been suggested that sleep deprivation (SD) may alter clock gene expression indirectly, through increased food intake. Although plausible, this notion is less straightforward than it seems. First, the effects of SD on clock gene expression are acute and can be observed already after 3 h of SD [9,10], which is inconsistent with the gradual re-entrainment over several days observed in food-restriction experiments. Second, whereas humans do eat more (and gain weight) when sleep deprived [12,13,14,15], it is unclear whether and, if so, how SD affects food intake acutely in the mouse. The aim of the current study was therefore two-fold. We first sought to establish whether access to food during SD affects the SD-induced changes in clock gene expression in the cerebral cortex. We then assessed whether, similar to humans, mice eat more when kept awake. We found that during SD, mice ate as much as during matching baseline hours and that the cortical clock gene expression changes after SD were unaffected by food deprivation. Moreover, despite normal food intake, mice lost weight during the SD and ate more during the subsequent recovery, demonstrating that the SD incurred a metabolic imbalance. We believe this imbalance to result from the metabolic cost of staying awake which we illustrate by putting food intake into a sleep-wake context.

## 2. Results

### 2.1. Sleep Deprivation Affects Clock Gene Expression Independent of Food Intake

To determine whether the SD-induced changes in the cortical expression of clock genes are caused by changes in food intake, we compared expression changes in the three core clock genes Per1, Per2, and Cry1 and the ‘clock-controlled’ gene Dbp in mice with and without access to food during a 6 h SD, starting at light onset (ZT0). Expression of the activity-induced short isoform of Homer1, Homer1a, considered a reliable marker of sleep homeostatic drive [16,17], was quantified in parallel. The mRNA levels reached after SD were contrasted to non-SD controls with or without access to food that were sacrificed at the same time of day (ZT6).

**Figure 1:**
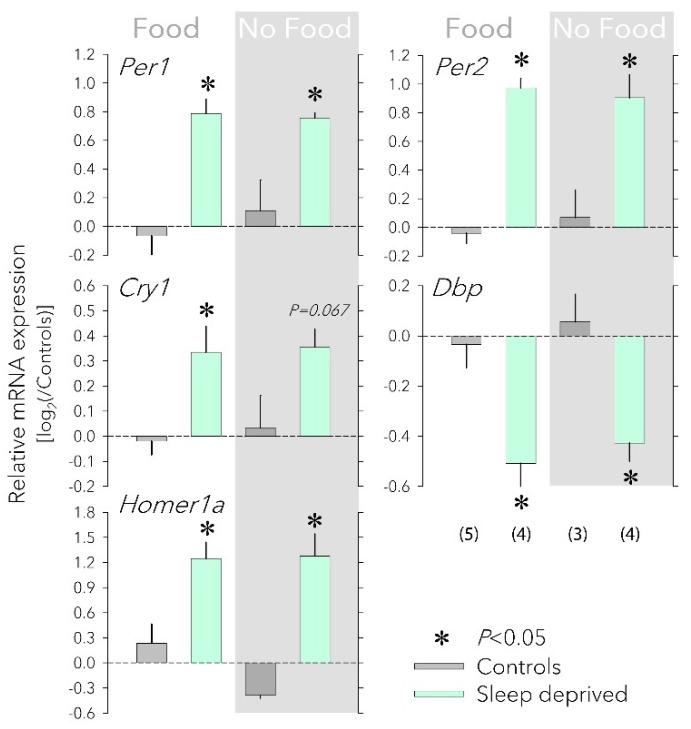
Effects of food availability on cortical clock gene expression. Having access to food (‘Food’, left) or not (‘No Food’, right, grey background) during a sleep deprivation (SD) did not affect the SD-induced changes in the cortical expression *Per1*, *Per2*, *Cry1*, *Dbp,* and *Homer1a*. Values for each transcript were first expressed relative to the expression of three housekeeping genes (*Eef1a*, *Gapdh*, and *Rsp9*) within each sample and subsequently to the mean of the two control groups (0 level, dark-grey bars) that each accompanied the SD (mint bars) of the two food conditions. Number of mice for each group in parentheses (error bars reflect 1 SEM). Food did not change the SD response (two-way ANOVA factor ‘Food’ *p* > 0.57, factor ‘SD’ *p* < 0.0021; interaction *p* > 0.17 for each of the five transcripts). Asterisks designate a significant SD effect within each food condition (two-sided post hoc *t*-tests; *p* < 0.05). mRNA levels of housekeeping genes were not affected by either SD or food access (not shown). All tissues were collected at ZT6.

In mice with access to food, SD elicited a robust increase in Homer1a, Per1, Per2, and Cry1 expression and decrease in Dbp expression (Figure 1), similar to what was previously shown [18]. Depriving mice of food during the SD did not modify these responses (Figure 1), demonstrating that increased food intake does not contribute to SD’s effect on clock gene expression.

### 2.2. Sleep Deprivation Does Not Alter Food Intake yet Animals Eat More during Recovery

**Figure 2:**
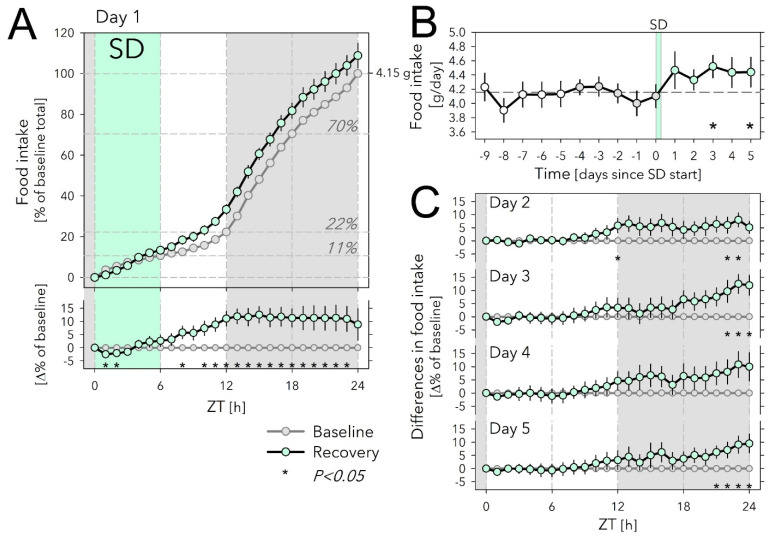
Effects of sleep deprivation on food intake. (**A**) Hourly values of food intake accumulated over 24 h and expressed as % of average daily food intake during the last 5 baseline days (Days −4 to 0; 100% = 4.15 g) preceding the sleep-deprivation (SD) day (Day 1). Food intake during SD (mint symbols and area) did not differ from baseline (grey symbols, 5-day averages). Horizontal lines in graph indicate % of total daily food eaten at consecutive 6 h intervals during baseline. Lower graph: SD-baseline difference in accumulated food-intake curves depicted in the upper graph. Gray area indicates the 12 h dark period. Asterisks designate hours with significant differences in food intake from baseline (paired two-sided *t*-tests; *p* < 0.05; *n* = 11–17; see Methods for *n*/day). (**B**) Daily food intake for the 10 days before and the 5 days after the SD. White symbols (Days −9 to −5) depict values for 5 additional baseline days recorded in cohort 1 (*n* = 6). Please note that these extra baseline days were not included in the analyses. Horizontal dashed line reflects mean over Days −4 to 0 (=100% in (**A**)). SD affected food intake (Days 1 to 5 vs. Days −4 to 0; two-way ANOVA factor ‘SD’ *p* = 0.004, ‘Day’ *p* = 0.89, interaction *p* = 0.78). Asterisks mark days with significant increase in food intake compared with baseline (average Days −4 to 0; paired two-sided *t*-tests; *p* < 0.05; *n* = 11–17; see Methods for *n*/day). (**C**) Recovery-baseline differences in accumulated hourly food-intake values for Days 2 to 5. As on Day 1, the increase in food intake on Day 2 was mainly due to extra food eaten during the 2nd half of the light phase, whereas for Days 3 to 5, extra food intake occurred at the end of the dark phase. Asterisks as in (**A**). Error bars mark ±1 SEM.

The idea that altered food intake might contribute to the SD-induced changes in clock gene expression is based on the assumption that animals eat more during SD. To test this assumption, we measured food intake using the *Phenomaster* feeding monitoring system, under baseline conditions (5 days prior SD; Days −4 to 0), during a 6 h SD (Day 1, ZT0–6), and during recovery (Day 1, ZT6–24 and Days 2 to 5; see Figure 2).

Under baseline conditions, mice ate 4.15 g chow per day, most of which was consumed during the 12 h dark phase (Figure 2A). Nevertheless, mice ate substantial amounts during the light phase as well, reaching 22.3% of daily food intake, half of which was consumed during the first 6 h (ZT0–6), i.e., the time the SD was scheduled on Day 1 (Figure 2A).

Surprisingly, although over the first 2 h of SD, mice ate somewhat less compared with matching baseline hours, when calculated over the entire 6 h SD, the amount consumed did not differ from baseline (13.3 vs. 10.7%; Figure 2A). Given this lack of an SD effect, it was perhaps even more surprising that over the first 6 h of recovery (Day 1, ZT6–12) they ate 73% more than baseline (20.1 vs. 11.6% of baseline daily food intake; Figure 2A) so that over the entire 12 h light phase, that included the SD, mice ate 0.45 g more than during baseline (+48%; 1.38 g vs. 0.93 g). This increase in accumulated food intake was maintained throughout the 12 h dark period of Day 1 because mice ate the same amount of food as in the preceding baseline dark periods. As a result, by the end of Day 1, SD mice had eaten 4.51 g, i.e., 9% more than the average daily food intake (Figure 2A).

Remarkably, daily food intake remained upregulated over the next 4 recovery days (Days 2 to 5), although statistical significance was not reached for Day 4 (Figure 2B,C). Interestingly, the time of day at which the extra food was consumed changed over the 5 recovery days; although during Day 2, similar to Day 1, animals ate more in the second half of the light phase, on subsequent days this occurred towards the end of the dark periods (ZT21–24 on Days 3 to 5; Figure 2C). During the first 9 h of the light periods (ZT0–9), food intake was remarkably constant among Days 2 to 5 and did not deviate from the baseline (Figure 2C).

### 2.3. Sleep Deprivation Dissociates Food Intake from Time Spent Awake

Knowing that extended wakefulness represents a metabolic burden [19,20,21], combined with our current observation that mice eat more after the SD but not during the SD, suggests that depriving mice of sleep incurred a metabolic deficit or, in other words, that per unit of time spent awake during SD, food intake was insufficient. To investigate this further, we quantified the relationship between time spent awake and food intake during baseline, SD, and recovery sleep. Although for technical reasons we could not record EEG/EMG and food intake simultaneously in the same individuals, we have abundant normative sleep-wake data of mice of the same inbred strain, sex, and age submitted to the same experimental protocol and conditions, to allow group-level comparisons. Here, we used published data of Diessler, Jan et al. [22] and Hor, Yueng et al. [23].

**Figure 3:**
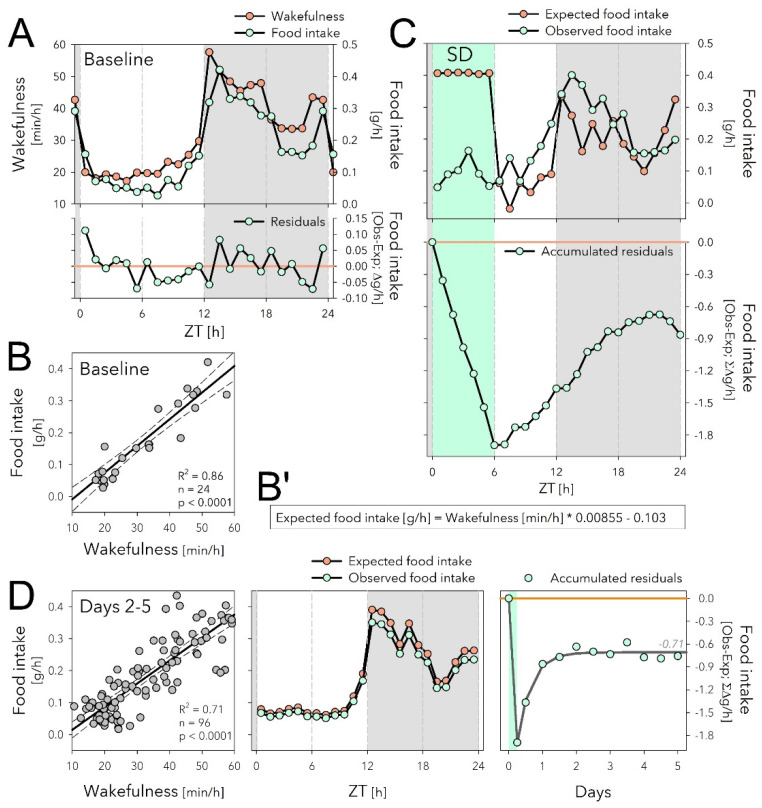
The sleep deprivation (SD) evoked dissociation between food intake and time spent awake might underlie increased food intake during recovery. (**A**) The time course of hourly values of food intake in baseline (mint symbols; mean over Days −4 to 0; same data as in Figure 2A), closely follows that of time spent awake (orange symbols). (**B**) Mean hourly values of wakefulness and food intake strongly correlated, with only small residuals that did not systematically vary with time of day (lower graph in (**A**)). (**B’**) Equation of the linear regression in panel B used to predict expected food intake in panels (**C**,**D**) (middle) based on time spent awake. (**C**) Given the equations in (**B’**), the expected food intake can be calculated for Day 1 including the SD (upper graph). SD induced a disassociation between food intake and time spent awake and animals accrued a deficit of 1.89 g of food intake during the SD that reduced to 0.86 g by the end of Day 1 (lower graph). (**D**) During Days 2 to 5 after SD, the relationship between waking and eating was highly correlated as in baseline (left panel, compare with (**B**)), and wake-derived expected food intake (given equation (**B’**)) predicted remarkably well the observed data (average over the 4 days, middle panel). The 0.86 g food deficit estimated for Day 1 (see (**C**)), was maintained during the following 4 days and stabilized at −0.71 g (right panel; non-linear regression, exponential saturating function fitted to 12 h values).

In baseline conditions, the time course of mean hourly values of food intake was tightly correlated with the time spent awake accounting for 86% of the variance in food intake (Figure 3A,B; adjusted R^2^ = 0.86; *p* < 0.0001, *n* = 24 1 h intervals). Moreover, the residuals of the linear regression did not systematically vary with time of day (Figure 3A, lower graph), suggesting that mice ate the same amount of food per unit of time awake, independent of time of day.

Using the relationship between food intake and time spent asleep established for the baseline (Figure 3B’), we then predicted expected food intake based on the sleep-wake values observed for Days 1 to 5 (Figure 3C,D). This analysis revealed a profound dissociation between time spent awake and food intake during SD; although mice were expected to eat 0.41 g when awake for 60 min in baseline conditions (Figure 3A,B’), the observed average hourly food intake during SD was 0.09 g; i.e., 4.6-fold lower than expected (Figure 3C, upper graph). The discrepancy between expected and observed food intake amounted to a total deficit of 1.89 g food intake when accumulated over the 6 h SD (Figure 3C, lower graph). This deficit of feeding relative to time spent awake was reduced to 0.86 g by the end of Day 1 because during the 18 h of recovery mice both ate more (+0.52 g; Figure 2A) and slept more (+2.04 h) than during the corresponding baseline period (Figure 3C, lower graph).

During subsequent recovery days (Days 2 to 5), the baseline relationship between food intake and time spent awake was re-established and the wake-derived expected food intake predicted the observed values remarkably well (Figure 3D, left and middle panels). During Days 2 to 5, the 0.86 g loss of food intake estimated for Day 1 did not further substantially decrease, and stabilized at around 0.71 g (Figure 3D, right panel). This was despite the increase in food intake accumulated over these 4 days (+1.15 g; Figure 2B), perhaps in part because mice slept less (−89 min, corresponding to 0.66 g extra food required according to the equation in Figure 3B’).

### 2.4. Sleep Deprivation Causes Weight Loss

**Figure 4:**
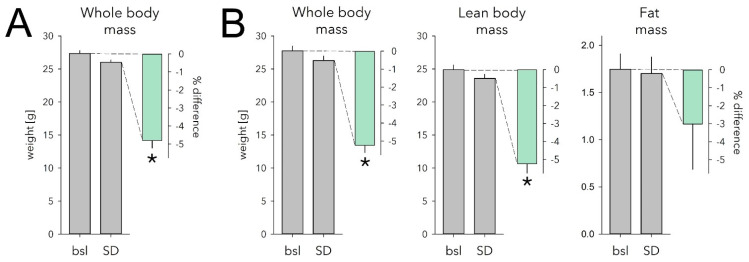
Effect of sleep deprivation (SD) on body weight and body composition. (**A**) SD caused a 4.8% weight loss in mice with ad libitum access to food (mint bar; *p* = 1.3 × 10^−5^, paired two-sided *t*-test, *n* = 9). Body weight measured after the 6 h SD (grey bar) ending at ZT6 was 1.3 ± 0.1 g lower compared with that reached at this time of day on two preceding baseline days (bsl; grey bar; average of the 2 days). (**B**) Body composition was measured in a separate cohort following the exact same protocol as in A. SD similarly reduced whole body weight in this cohort (−1.5 ± 0.1 g, 5.2%; *p* = 9.6 × 10^−5^, paired two-sided *t*-test, *n* = 6). Lean body mass decreased to the same extent (−1.3 ± 0.1 g, 5.2%; *p* = 3.1 × 10^−4^). Fat mass was reduced as well but not significantly so (−0.04 ± 0.04 g, 3.0%, *p* = 0.33). Details as in (**A**). * marks significant SD vs. bsl effects. Note different scale for grams of fat mass. Error bars mark 1 SEM.

The above analyses indicate that during SD, mice eat less than expected based on the time spent awake. This could represent an energy imbalance likely to underlie the increase in food intake we observed during the 6 h following the SD. The ‘eating-less-than-expected’ phenomenon could also lead to weight loss during the SD, which would provide more direct evidence documenting an energy deficit. To assess this, we measured each animal’s weight at the end of a 6 h SD at ZT6 and compared this with its baseline body weight reached at the same time of day measured 1 and 4 days prior to the SD. All animals were given ad libitum access to food. We found that SD indeed led to a highly significant 4.8% loss in body weight (−1.3 ± 0.1 g; paired two-sided *t*-test; *p* = 1.3 × 10^−5^; *n* = 9; Figure 4A), despite mice having free access to food.

To identify which body compartment (fat or lean body mass) accounted for this decrease in body weight, we next assessed the effect of SD on body composition in a separate cohort of mice. Consistent with the previous two cohorts, SD mice lost 5.2% body weight compared with baseline (−1.5 ± 0.1 g, *p* = 9.8 × 10^−5^, Figure 4B). This decrease was due mainly to a 5.2% loss of lean body mass (-1.3 ± 0.1 g, *p* = 3.1 × 10^−4^; Figure 4B). Fat mass was reduced as well, but not significantly so (−0.04 ± 0.04 g, 3.0%, *p* = 0.33; Figure 4B). Water loss did not seem to play a role as tissue hydration was not affected by SD (baseline: 85.3 ± 0.3% vs. SD: 84.8 ± 0.2%; *p* = 0.23; see Methods).

## 3. Discussion

The first main finding of our study is that having access to food or not during SD does not affect the SD-induced changes in cortical clock gene levels. This indicates that changes in clock gene expression during SD do not relate to increased food intake as observed in SD studies in humans. The second main finding is that, contrary to expectation, SD deprives mice of food. Although eating as much as during corresponding baseline hours and despite having free access to food, animals nevertheless lose 5% of their body weight when kept awake and eat more than baseline during the first 6 h of subsequent recovery. These observations show that the amount of food eaten during SD was insufficient to cover energy expenditure, leading to a metabolic deficit.

### 3.1. Sleep Deprivation or Food Deprivation?

As mice have a small body size they have high metabolic rates compared with humans [24,25]. C57BL/6J mice, at the body weights seen in our study (27–28 g), spent 51–57 kJ/day [26]. This energy expenditure (EE) has to be balanced by energy intake through food, which, estimated on the 4.15 g/day we observed and given the caloric content of the food (see Methods), amounts to 54 kJ/day. Corrigan et al. [26] also quantified the daily changes in EE, which as for food intake (Figure 3), matched the distribution of our wakefulness data remarkably well (Figure S1). From the relationship between hourly values of time spent awake and EE, one can estimate that the baseline time spent awake (i.e., ca. 13 h/day), compared with sleeping 24 h, adds 15 kJ, i.e., 27% of the daily EE.

Mice lost ca. 1.4 g body weight during the 6 h SD of which 1.3 g was lean body mass and 0.04 g fat. Using energy density estimates for fat and lean-body mass (39.5 and 7.6 kJ/g, respectively [27]), the 1.4 g weight loss we observed would produce 11.5 kJ used to offset the cost of being kept awake while not eating enough. Adding the energy from food intake during the SD (7.2 kJ), the available energy during the SD would have amounted to 18.7 kJ. According to the time spent awake to food intake correlation, mice were expected to eat 1.89 g more food during the SD than they did, 1.03 g of which was recovered by eating and sleeping more. Using these estimates, the incurred energy deficit would amount to 13.5–24.8 kJ, a range compatible with the above estimation based on the tissue energy estimates.

A similar, 1.2 g (5%) loss of body weight was observed after a 5 h food deprivation (FD) at approximately the same time of day (ZT23–4) [28]. In that study, the weight loss was due to a decrease of 0.2 g fat mass and of 0.8 g of lean body mass which would represent 14 kJ of energy. Although these remain back-of-the-envelope calculations and the actual metabolic cost associated with an SD should be assessed experimentally, both the FD and SD study do demonstrate that short-term decreases in food intake at a time of day animals normally eat little, can have important consequences on physiology and behavior. Besides weight loss, both interventions share a number of other physiological consequences such as increased levels of corticosterone and amylase and metabolites such as carnitines [22,29,30,31,32]. Sleep was not quantified in the FD study, and it is therefore not possible to assess whether its effects are solely due to reduced energy intake or also because of increased energy expenditure resulting from increased time spent awake related to food-seeking behavior and/or stress [33,34]. Similarly, the consequences of SD that sleep researchers attribute to the incurred sleep loss, might instead be due to deficits in food intake. For example, the time spent awake to food intake correlation illustrates how the well-known rebound in sleep time after SD can be put into an energy context as, together with eating more, it appears an efficient measure to offset the SD-incurred energy deficit.

We found food intake to be increased, compared with baseline, during the days after the SD, pointing to potential long-term effects on energy balance. Alternatively, the slight decrease in sleep time over these 5 recovery days and/or normal growth might have contributed to higher energy intake. We previously reported on long-term effects on the brain transcriptome notably on the expression of circadian clock genes [23]. Given that clock genes and energy metabolism are tightly coupled [35,36], these observations point to a hereto unnoticed and surprisingly long-lasting effects on energy metabolism after only a single, short-term perturbation of sleep. Long-lasting electrophysiological consequences of a 6 h SD have also been observed in the rat hypothalamus, including the arcuate nucleus [37], which is important in regulating food intake and energy balance [38].

### 3.2. Circadian, Sleep-Wake, or Food Driven?

An obvious and intriguing question is why mice allow body weight to drop instead of simply eating more during the SD. This is also unexpected because acute SD in the rat signals hunger considering the rapid increase in ghrelin during SD, especially in the hypothalamus [39]. SD also increases the physiological signals of hunger in humans indicated by the increases in ghrelin and decreases in leptin [12] but in contrast to mice, humans do respond to these signals by eating more than usual [13,14,15]. Upon food deprivation ghrelin activates the agouti-related protein (AgRP) expressing neurons in the arcuate nucleus, which are important for regulating food intake and food-seeking behavior [40,41]. Accordingly, optogenetic stimulation of these neurons in mice promoted wakefulness during which they ate and displayed food-seeking behavior [41]. The same study showed that SD attenuated these behavioral effects of stimulating AgRP-positive neurons, suggesting a potential mechanism underlying the dissociation we observed between wakefulness and food intake during SD.

Circadian factors and/or the presence of light might actively inhibit food intake during the rest phase. Food restriction experiments in which access to food is restricted to this inappropriate circadian phase indeed show that mice only slowly adapt to such paradigms and initially lose weight [42]. The increase in food intake observed in the remainder of the light phase (ZT6–12) demonstrates, however, that mice, when hungry, are able to eat more at this circadian phase and in the presence of light. The increased food intake at this time is all the more surprising as it competes which sleep’s homeostatic drive which is considered to be greatest immediately after the SD. During the subsequent dark phase, the circadian phase when mice habitually eat the most, food intake no longer differed from the baseline but the rebound in sleep time was largest [43]. This increase in sleep time can also be seen as a behavioural measure to balance the energy budget. Alternatively, as the SD protocol is considered a mild stressor [44], stress might have kept mice from eating more. The mere presence of the experimenter might interfere with feeding behavior. Whether circadian time, light, or SD-associated stress interfered with proper feeding during the SD could be assessed by sleep depriving mice in the dark period. Although during this period SD also increased corticosterone levels [45], circadian phase and the absence of light would favour feeding.

The circadian clock, located in the SCN, drives the daily changes in the sleep-wake distribution. Additionally, food intake and EE are considered to be under strong and direct circadian control [36,46]. Our correlation analyses demonstrated, however, that the changes in food intake result from changes in time spent awake as food intake expressed per minute of wakefulness does not appreciatively vary with time of day. Similarly, EE closely tracks time spent awake [47] (Figure S1 [26]). This tight relationship between waking, EE, and food intake, makes sense given the high metabolic rates of mice because, as we show here, the extra energy spent by being awake requires immediate refuelling to avoid deficits in energy balance.

Clock genes engage in transcriptional-translational feedback loops (TTFL) that constitute the molecular time-keeping circuitry underlying circadian rhythm generation [48]. Besides being part of the circadian TTFL circuitry, several core clock genes act as sensors of environmental and systemic signals [5,49,50,51,52,53]. For example, the clock gene *Per2* can be rapidly induced by stress, light, changes in temperature, and bloodborne systemic cues [54,55,56,57,58]. As a consequence, in tissues peripheral to the SCN such as the cerebral cortex, *Per2* expression is determined by both circadian and sleep-wake driven factors [7,11,18,59]. Given our current results, the acute SD-incurred changes in clock gene expression clearly do not result from eating more. Instead, it can be argued that clock gene expression in the cortex responds to the rapid changes in metabolic drive associated with wakefulness both under baseline and SD conditions. In this context, the increase in *Per2* expression observed under conditions of food restriction might be associated with the waking preceding to the time window of food accessibility window (i.e., food anticipatory activity) and not by food intake per se. To address this experimentally and to disassociate increased wakefulness from the energy deficit imposed by SD, additional experiments are required, involving offering more palatable food or artificial feeding during SD.

In summary, our study shows that in mice a one-time, short sleep deprivation leads to an immediate caloric deficit that resulted in an immediate loss of body weight. This finding contrasts observations in humans documenting an increase in food intake during sleep deprivation and associated weight gain. This species difference might relate to factors such as stress experienced during the SD in mice. The notion that in mice a sleep deprivation also represents a food deprivation puts the consequences of this intervention, which are commonly attributed to the loss of sleep, in a new perspective. For example, recovery sleep in mice could not only be seen as a response to correct for sleep time lost but also as a behavioral strategy to balance the body’s energy budget. The relationship between sleep and energy balance might be bi-directional, as sleep homeostatic pressure appeared reduced in mice kept under a food-restriction protocol, possibly to facilitate food-seeking behavior [41,60].

## 4. Materials and Methods

### 4.1. Animals and Housing Conditions

All mice were male C57BL/6J mice, aged 10–16 weeks at the time of recording and were individually housed during the recordings under 12 h light/12 h dark conditions at 23 °C ambient temperature. Food (standard chow: 3436 Kliba Nafag, Switzerland with a 13.1 kJ/g metabolizable energy content) and water were given ad libitum. All animal procedures followed the guidelines of Swiss federal law and were pre-approved by the Veterinary Office of the Canton of Vaud.

### 4.2. Keeping Mice Awake

Sleep deprivations (SD) were performed between zeitgeber time (ZT)0, i.e., light onset, and ZT6, i.e., the mid-point of the 12 h light phase. During this period, mice usually spent most of their time asleep. Mice were kept awake using the gentle handling method [61], which is somewhat of a misnomer as mice were not handled by the experimenters. Instead, mice remained in their home cage with free access to food and water and were left undisturbed unless signs of sleep appeared. In these instances, the experimenters, who were present in the animal room for the duration of the SD, prevented sleep by introducing and again removing paper tissue, changing the litter, bringing a pipet in the animal’s proximity, or gentle tapping of the cage.

### 4.3. Quantification of Cortical Gene Expression

Four experimental groups of mice were used: (i) control mice with ad libitum food and sleep (*n* = 5), (ii) mice kept awake (SD; ZT0−6) with free access to food (*n* = 4), (iii) control mice with ad libitum sleep but no access to food between ZT0−6 (*n* = 3), and (iv) mice with no access to sleep or food between ZT0−6 (*n* = 4).

At ZT6, mice of all 4 groups were anesthetized with isoflurane and immediately sacrificed by decapitation. Cerebral cortex tissue was quickly dissected, immediately frozen in liquid nitrogen, and then stored at −80 °C. Total mRNA was extracted from 30 mg of cortical tissue using the RNeasy Mini kit (Qiagen, Düsseldorf, Germany) following the manufacturer’s instructions. Briefly, each sample was homogenized by pestle using 350 µL of RLT buffer (supplemented with β–mercaptoethanol from Sigma-Aldrich instead of DTT) on ice and passed on a QIAshredder column (Qiagen, Düsseldorf, Germany). Contaminating DNA was removed with DNase I (Qiagen, Düsseldorf, Germany). RNA quantification was performed on a Nanodrop ND1000 (Marshall Scientific, Hampton, NH, USA) with a consistent 260/280 ratio between 1.8 and 2.1. Reverse transcription from 500 ng of RNA was performed using the SuperScript II Reverse Transcriptase (Invitrogen, Carlsbad, NM, USA) following the manufacturer’s instructions. cDNA was diluted 10 times in DNAse free water. Reactions without enzyme (NEC) and without the template (NTC) were performed in parallel as negative controls.

qPCR was performed according to the Applied Biosystems protocol using a 7900HT Fast Real-time (Thermo Fisher, Waltham, MA, USA) PCR system with SDS 2.3 software (Applied Biosystems, Foster City, CA, USA). For each reaction, 7.2 µL of the diluted cDNA was supplemented with 18.0 µL FastStart Universal Probe Master 2x (Roche Diagnostic GmbH, Mannheim, Germany), 0.90 µM of primer and 0.25 µM of probe for a total reaction mix of 36.0 µL. The qPCR was performed under standard cycling conditions (50 °C for 2 min, 95 °C for 10 min, followed by 45 cycles of 95 °C for 15 s and 60 °C for 1 min). Each qPCR reaction was performed in triplicate. The oligonucleotide sequences used can be found in Supplementary Table S1. Primers and probes were purchased from Invitrogen or Eurogentec and all probes were dual labelled with 5′FAM/3′BHQ as fluorophore and quencher. *Ribosomal protein S9* (*Rsp9*), *eukaryotic translation elongation factor 1A* (*Eef1a*), and *glyceraldehyde-3-phosphate dehydrogenase (Gapdh)* were found to be appropriate control genes to normalize qPCR experiments following the method proposed by Vandesomplele et al. [62]. Although SD affects the cortical expression of almost all known clock genes, not all react immediately at the end of SD [23]. We chose *Per1*, *Per2*, *Cry1*, and *Dbp* because their expression is known to change at that time point [10,18]. The relative quantification of the expression of these clock genes and of *Homer1a* was calculated with a modified ΔΔCt method [63]. All stability calculations, normalization, and quantification were performed using *qbase^PLUS^ 1.5* (Biogazelle, Machelen, Belgium). For displaying purposes, gene expression levels were expressed relative to the mean levels reached in the two sleep control groups (i.e., groups i and iii). Gene expression in the control groups was not affected by having access to food or not (post hoc *t*-tests: for *Per1*, *Per2*, *Cry1*, and *Dbp p* > 0.47; for *Homer1a p* > 0.09). Effects of SD were assessed by comparing the levels reached after SD to levels obtained in controls (i.e., groups ii and iv vs. groups i and iii, respectively).

### 4.4. Quantification of Food Intake

Food intake was recorded in two separate cohorts of mice (*n* = 6 and 11, respectively). Mice of both cohorts were habituated to the recording cages for at least 11 days prior to the sleep deprivation (designated as Day 1 starting at light onset). In cohort 1, baseline food intake was measured continuously for 10 days (Days −9 to 0). On the following day (Day 1), mice were sleep-deprived between ZT0 and −6. The remaining 18 h of Day 1 and Day 2 were considered recovery. On Day -2 recording had to be restarted and data of the entire day were discarded for all mice. Additionally, data of Day 0 had to be discarded due to technical problems. In cohort 2, the baseline was recorded for 5 days (Days −4 to 0), followed by Day 1 (6 h SD + 18 h recovery) and 4 more recovery days (Days 2 to 5). One mouse could only be recorded until dark onset (ZT12) of Day 1; i.e., the SD plus the first 6 h of recovery, due to technical problems. Combined, the 2 cohorts spanned 10 days of baseline and 5 days of recovery (including the SD) with the following numbers of mice contributing to each day: Days -9 to 0: *n* = 6, 6, 6, 6, 6, 17, 17, 11, 17, 11; Days 1–5: *n* = 17/16, 16, 11, 11, 11. To construct a baseline food intake time course, data were collapsed into hourly intervals and then averaged over the 5 days preceding SD (Days −4 to 0; i.e., for cohort 1: Days −4, −3, and −1; cohort 2: Days −4 to 0). To assess the effects of SD on food intake during SD and recovery each animal served as its own control (i.e., SD vs. baseline).

Food intake was quantified as food disappearance from the feeders with the *Phenomaster* feeding-monitoring system (TSE Systems GmbH, Bad Homburg, Germany), located in a dedicated room with restricted access to minimize the impact of human presence. Mice were individually kept in standard housing conditions (see above) with food and water ad libitum in cages equipped with rat-type feeders with large food and spillage containers to minimize maintenance during experiments. Feeder scales were calibrated according to the manufacturer’s instructions using 4 weights (range: 0–90 g). The sensitivity of measurement was 10 mg. Food intake was regularly monitored throughout the experiments from remote using TeamViewer (TeamViewer AG, Göppingen, Germany).

Food disappearance was collected at 1 min resolution as unfiltered, true feeder-weight values with the following analogue-to-digital converter (ADC) settings: smoothing *ADC*: 5 s, *trial monitor observation interval*: 1 s, and *Max Delta ADC*: 80. To assess the quality of raw data, in-software filtering was disabled and was instead applied after acquisition and inspection of the raw data using Excel(Microsoft, Redmond, WA, USA). Artefacts (mostly sudden positive or negative deflections related to mice touching the feeders) were removed for data points with values differing by more than −0.15 or +0.5 g relative to that of the preceding data point by setting their values to that of the preceding data point, following the filter settings of the manufacturer’s software.

Food spillage not accounted for by the feeding system was assessed in a pilot experiment using special bedding (Lignocel^®^ nesting small; JRS, Rosenberg, Germany) that, upon removal from the cage, reveals spillage at the cage bottom. Spillage was fully contained within the feeder as we found no measurable spillage on the cage bottom after the 3-day test. We estimate in-cage spillage to be <1%.

### 4.5. Quantification of Sleep

Previously published sleep–wake data [22,23] were used to relate hourly mean values of time spent awake to hourly food intake values measured in the current study. Male C57BL/6J mice 10–12 weeks (*n* = 12) at the time of SD were kept under our standard housing conditions (see above). EEG/EMG was recorded continuously from 4 days; 2 baseline days, followed by a 6 h SD starting at ZT0 and 42 h of recovery (Days 1 and 2). In 6 of these mice, recovery was recorded for an additional 5 days.

To determine sleep–wake states, mice were equipped with chronic EEG and EMG electrodes under deep anesthesia according to methods detailed in [61]. The electrode leads were connected to a socket fixed to the skull to which the recording lead could later be fitted. The head assembly prevented easy access to food in the feeder and thus mice in which food intake was monitored the EEG/EMG could not be recorded at the same time. A Xylazine (10 mg/kg)/Ketamine (100 mg/kg) mix was injected IP ensuring a deep plane of anesthesia for the duration of the surgery (ca. 30–40 min). Analgesia was provided the evening prior and the 3 days after surgery (Dafalgan in drinking water; 200–300 mg/kg). Mice were allowed to recover for at least 10 days prior to baseline recordings. Electrophysiological signals were captured and ADC at (sampling rate 2000 Hz, down-sampled and stored at 200 Hz) using *Embla A10* and *Somnologica-3* hard- and software, respectively (Medcare Flaga; Thornton, CO, USA). The sleep–wake states REM sleep, NREM sleep, and wakefulness were annotated for consecutive 4 s time windows based on the EEG and EMG patterns according to established criteria [61]. Four-second time windows scored as wakefulness were collapsed into hourly values and expressed as min/h. The two baselines were averaged to generate a single 24 h time course.

### 4.6. Quantification of Body Weight and Body Composition

Male C57BL/6J mice, aged 13–14 weeks, were kept under our standard housing conditions (see above). The effect of SD on body weight was assessed in two cohorts (*n* = 5 and 4, respectively). Each animal was individually housed 10 days before SD and weighed between ZT6.0−6.5 on 3 days, i.e., 4 days and 1 day before the SD and on the SD day itself. SD was scheduled from ZT0-6 as in all other experiments. The first 2 measurements were averaged and considered as baseline to which body weight after the SD was contrasted within individual mice. The two baseline measures did not differ (−0.14 ± 0.10 g; two-sided paired *t*-test, *p* = 0.21, *n* = 9). For weighing, animals were transported and measured while in a cardboard tube to reduce handling stress. Balance gave weights at 0.01 g precision.

Following the same experimental protocol used for the body weight assessment explained above, in a 3rd cohort (male C57BL/6J mice, aged 13–14 weeks, *n* = 6), body composition was assessed using an EchoMRI™ 3-in-1 (Houston, TX, USA). Body weight, fat mass, lean body mass, and free and total water content were quantified. Hydration percentages were calculated as follows: (total–free water) * 100/lean body mass. Mice were lightly anesthetized with isoflurane before putting them into the analyzer. The whole procedure (weighing, induction of anesthesia, data acquisition) did not take more than 5 min. Measures of body weight, and body composition (% hydration, fat mass, and free and total water) did not differ between the two baseline measures (*p* = 0.29, 0.59, 0.65, 0.77, and 0.06, respectively), with the exception of lean body mass (−0.41 ± 0.11 g, 2nd vs. 1st baseline day; two-sided paired *t*-tests, *p* = 0.014, *n* = 6).

### 4.7. Statistics

For data organization, filtering, and collapsing into hourly values, Excel (Microsoft, Redmond, WA, USA) and TMT Pascal (version 5.01, Framework Computers Inc., Brighton, MA, USA) were used. Statistics were performed in *SAS* (version 9.4), linear and non-linear regression analyses in SigmaPlot (version 12.5, Systat Software Inc., San Jose, CA, USA). SigmaPlot was also used to generate the graphs. As the threshold of significance, *α* = 0.05 was used. Results are given as mean values ± SEM. Details on a number of biological (and technical) replicates per experiment are stated in Methods. Gene expression was analyzed first with two-way analysis of variance (ANOVA) with factors ‘Food’ and ‘SD’. Post hoc contrasts were analyzed using two-sided *t*-tests. Effects of SD on daily food intake during recovery days (Days 1 to 5) compared with baseline (Days −4 to 0) was analyzed with a two-way ANOVA with factors ‘Day’ and ‘SD’ followed by post hoc paired two-sided *t*-tests assessing deviations from the individual baseline means. Food intake differences between corresponding accumulated hourly values of SD and recovery (Days 1 to 5) and baseline (mean Days −4 to 0) were analyzed with paired two-sided *t*-tests, as were the effects of SD on body weight and body composition.

## Data Availability

Data are available upon request.

## Supplementary Materials

The following are available online at https://www.mdpi.com/article/10.3390/clockssleep4010006/s1, Figure S1: Relationship between energy expenditure (EE) and time spent awake under baseline conditions, Table S1: Primers and probes used for quantitative PCR.
