# Supplementary material for: Depriving Mice of Sleep also Deprives of Food
Source: Clocks Sleep. 2022 Feb 11;4(1):37–51. doi: 10.3390/clockssleep4010006 (PMC8884003; doi:10.3390/clockssleep4010006)
Supplement: Supplementary file 1 [file clockssleep-04-00006-s001.zip › clockssleep-1544996-supplementary.pdf]

Supplementary files

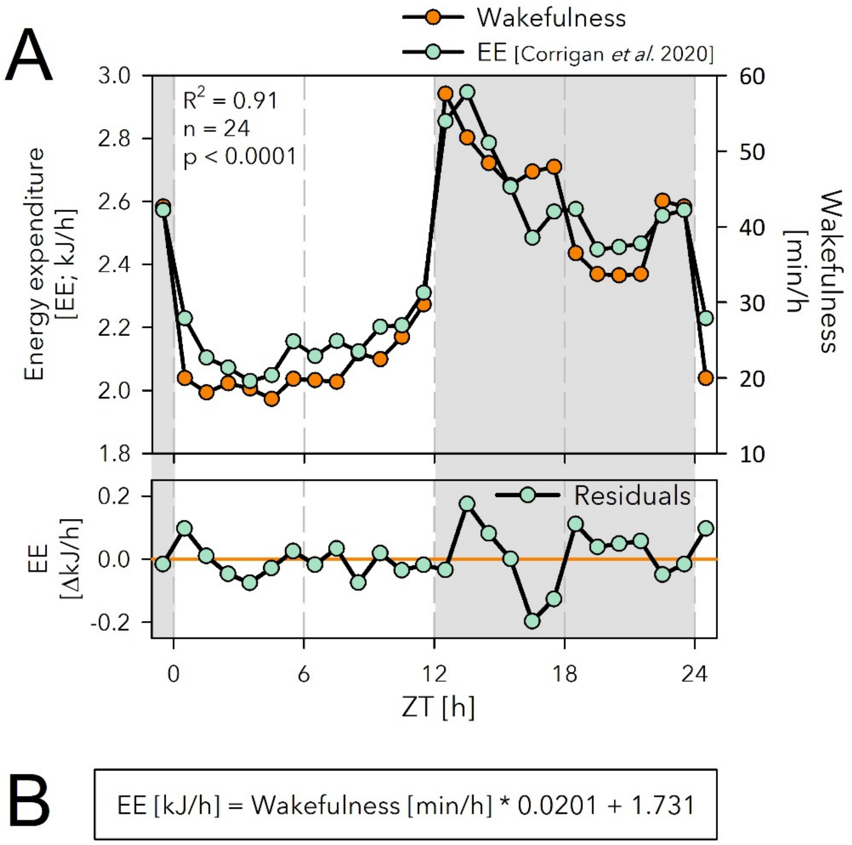

Figure S1: Relationship between energy expenditure (EE) and time-spent-awake under baseline conditions. (**A**) The time course of hourly values of EE (mint symbols; mean over 3 days; *n*=8, body weight: 28.4g, C57BL/6J males; data taken from Corrigan *et al.* 2020) closely follows that of time-spent-awake (orange symbols, same as in Fig. 3A). The two variables strongly correlated (linear regression) with only small residuals that did not systematically vary with time-of-day (lower graph). (**B**) Equation of the linear regression, which predicts that a mouse will spent 1.73 or 2.9kJ, if asleep or awake for 60min, respectively, in a given hour.

Gene		Sequence (5' --> 3')	Accession
Eef1a1	*Forward*	CCT GGC AAG CCC ATG TGT	NM_010106.2
	Reverse	TCA TGT CAC GAA CAG CAA AGC	
	Probe	TGA GAG CTT CTC TGA CTA CCC TCC ACT TGG T	
Gapdh	*Forward*	TCC ATG ACA ACT TTG GCA TTG	NM_001001303.1
	Reverse	CAG TCT TCT GGG TGG CAG TGA	
	Probe	AAG GGC TCA TGA CCA CAG TCC ATG C	
Rps9	*Forward*	GAC CAG GAG CTA AAG TTG ATT GGA	NM_029767.2
	Reverse	TCT TGG CCA GGG TAA ACT TGA	
	Probe	AAA CCT CAC GTT TGT TCC GGA GTC CAT ACT	
Per1	*Forward*	ACC AGC GTG TCA TGA TGA CAT AC	NM_011065.4
	Reverse	CTC TCC CGG TCT TGC CTT CAG	
	Probe	CCG TCC AGG GAT GCA GCC TCT	
Per2	*Forward*	ATG CTC GCC ATC CAC AAG A	NM_011066.3
	Reverse	GCG GAA TCG AAT GGG AGA AAT	
	Probe	ATC CTA CAG GCC GGT GGA CAG CC	
Cry1	*Forward*	GTT GGC CGG CTC TTC CA	NM_007771.3
	Reverse	CAA GAT CCT CAA GAC ACT GAA GCA	
	Probe	CGT GGG CAT CAA CAG GTG GCG	
Dbp	*Forward*	CGT GGA GGT GCT TAA TGA CCT TT	NM_016974.3
	Reverse	CAT GGC CTG GAA TGC TTG A	
	Probe	AAC CTG ATC CCG CTG ATC TCG CC	
Homer1a	*Forward*	GCA TTG CCA TTT CCA CAT AGG	NM_011982.2
	Reverse	ATG AAC TTC CAT ATT TAT CCA CCT TAC TT	
	Probe	ACA CAT TCA ATT CAG CAA TCA TGA	

Table S1: Primers and probes used for quantitative PCR
